# Artificial intelligence in diabetic retinopathy screening: clinical assessment using handheld fundus camera in a real-life setting

**DOI:** 10.1007/s00592-023-02104-0

**Published:** 2023-05-08

**Authors:** Marco Lupidi, Luca Danieli, Daniela Fruttini, Michele Nicolai, Nicola Lassandro, Jay Chhablani, Cesare Mariotti

**Affiliations:** 1 Eye Clinic, Department of Experimental and Clinical Medicine, Polytechnic University of Marche, Ancona, Italy; 2 Fondazione per la Macula Onlus, Dipartimento di Neuroscienze, Riabilitazione, Oftalmologia, Genetica e Scienze Materno-Infantili (DINOGMI), University Eye Clinic, Genoa, Italy; 3 IRCCS - Fondazione Bietti, Rome, Italy; 4 Department of Medicine and Surgery, University of Perugia, S. Maria della Misericordia Hospital, Perugia, Italy; 5 Department of Ophthalmology, UPMC Eye Center, University of Pittsburgh, Pittsburgh, USA

**Keywords:** Diabetic retinopathy, Artificial intelligence, Screening, Handheld fundus cameras

## Abstract

**Aim:**

Diabetic retinopathy (DR) represents the main cause of vision loss among working age people. A prompt screening of this condition may prevent its worst complications. This study aims to validate the in-built artificial intelligence (AI) algorithm Selena+ of a handheld fundus camera (Optomed Aurora, Optomed, Oulu, Finland) in a first line screening of a real-world clinical setting.

**Methods:**

It was an observational cross-sectional study including 256 eyes of 256 consecutive patients. The sample included both diabetic and non-diabetic patients. Each patient received a 50°, macula centered, non-mydriatic fundus photography and, after pupil dilation, a complete fundus examination by an experienced retina specialist. All images were after analyzed by a skilled operator and by the AI algorithm. The results of the three procedures were then compared.

**Results:**

The agreement between the operator-based fundus analysis in bio-microscopy and the fundus photographs was of 100%. Among the DR patients the AI algorithm revealed signs of DR in 121 out of 125 subjects (96.8%) and no signs of DR 122 of the 126 non-diabetic patients (96.8%). The sensitivity of the AI algorithm was 96.8% and the specificity 96.8%. The overall concordance coefficient k (95% CI) between AI-based assessment and fundus biomicroscopy was 0.935 (0.891–0.979).

**Conclusions:**

The Aurora fundus camera is effective in a first line screening of DR. Its in-built AI software can be considered a reliable tool to automatically identify the presence of signs of DR and therefore employed as a promising resource in large screening campaigns.

## Introduction

As a well-known microvascular complication of diabetes, diabetic retinopathy (DR) represents the main cause of vision loss and preventable blindness among the working age adults (20–74 years) [1]. According to a recent meta-analysis the prevalence of type 2 diabetes is increasing in both low-income and middle-income countries in the years between 1990 and 2015, causing blindness in 0.4 million people [2]. Even in Europe the number of people with diabetes affected by any eye disease is estimated to increase from 6.4 million in 2019 to 8.6 million in 2050 [3]. In such an evolving scenario a correct and effective screening among diabetic people is an essential tool for identifying early stages of DR and correctly address them toward the best therapies. DR screening strategies landscape is changing very rapidly, thanks to new technologies and, even due to coronavirus disease 2019 (COVID-19) pandemic, the development of telemedicine, artificial intelligence technologies together with handheld portable devices for retinal imaging is progressively imposing with good image quality and a diagnostic accuracy comparable to that given by traditional tabletop fundus cameras [4]. Another advantage of handheld fundus cameras is the portability that enable operators to reach patients even in rural contexts and out of traditional settings of ophthalmological screening.

With the help of artificial intelligence (AI), an automated image analysis can help referral of DR at an early stage of the disease [5]. All these characteristics are present in the Optomed Aurora IQ camera.

Aim of this study is to validate the AI deep learning algorithm Selena+ of the handheld Aurora fundus camera (Optomed, Oulu, Finland) for a first line screening in a real-life setting.

## Methods

### Population

This was a prospective, observational cross-sectional study, compliant with the tenets of the Declaration of Helsinki. Informed consent was obtained by each patient prior to the study enrollment. Patients were recruited from those referred between January 2022 and April 2022 to the Medical Retina Service of the Eye Clinic of Azienda Ospedaliero-Universitaria Ospedali Riuniti of Ancona (Italy).

Inclusion criteria were: patients aged > 18 years both affected or not by diabetes. Exclusion criteria were: history of any ophthalmologic disease that could affect the retinal aspect differently from DR (e.g., any other maculopathy, uveitis, congenital ocular malformations) or that could impair adequate fundus visualization (e.g., vitreous hemorrhage, clinically significative cataract [> 3G] or other media opacities), patients who showed poor cooperation.

### Image acquisition

Each patient underwent an acquisition of fundus photography with the Aurora handled fundus camera after having rest for a few minutes in a dark room, that enabled us not to use any mydriatic eyedrop in this phase.

The Optomed Aurora IQ camera is a handheld non-mydriatic digital fundus camera that permit the acquisition of 50° high resolution (2368 × 1776 pixels, 300 dpi) color images. The camera dispose of both a manual focus (correction from − 20 to + 20 diopters) and auto-focus (correction from − 15 to + 10 diopters) and autoexposure, which allows the user to automatically adapt the brightness of the image to the patient’s eye; in our study we applied the auto-focus and auto-exposure settings. The photo acquisition is possible even in myosis because the minimum pupil diameter needed to obtain good quality images is of 3.1 mm. The Optomed Aurora IQ camera is able to provide an AI image analysis services over Internet access. This is enabled by registering the camera for the use of Optomed provided AI service. Result of AI analysis is shown in the camera display after imaging or can be visualized in a second time by using the Avenue Archive software (Optomed, Oulu, Finland).

The image acquisition protocol consisted in a single 50° color photo cantered on the patient’s macula. All images were acquired by a single operator (L.D.).

### Image analysis and ophthalmological examination

After this phase, all images were analyzed by a single blinded operator (L.D.) using a 24 inches high-resolution TV screen for identifying the presence of ophthalmological signs of DR and classified as pathological and non-pathological according to the International Clinical DR Classification System [6]. Then the patients continued a complete ophthalmological examination including best corrected visual acuity, Goldman applanation tonometry and fundus examination after pupil dilatation by an experienced blinded operator (M.L). The fundus biomicroscopic examination was aimed to classify the retinal status as pathological or non-pathological according to the above-mentioned criteria.

### AI analysis

All images obtained with the Aurora camera were then analyzed with the AI image analysis service supported by Avenue Archive. Avenue Archive is a Windows-based software used for storing images and provide an automated AI-based assessment of the images taken with Optomed Aurora IQ fundus camera. For study purposes, images were transferred to the Avenue Archive software from the camera and analyzed not immediately after the acquisition. Nevertheless, the Aurora IQ fundus camera has the direct access to the AI algorithm Selena+ for DR screening and the images can be assessed almost in real-time after the acquisition (mean processing time is 5 s). The access to the Optomed provided AI service is secured by an authentication process and encryption. The AI service is anonymous, meaning that it does not receive or store patient data. The results given by this AI algorithm classify images as pathological and non-pathological. In case of positive response, a disease staging of DR is also provided, however, in this study we decided not to evaluate this function since the study was designed for screening purposes. The algorithm has not the possibility to investigate and grade separately Diabetic Maculopathy, for this reason we decided not to take in consideration this parameter while grading color photo.

### Statistical analysis

The diagnostic accuracy of each DR detection assessment was computed using sensitivity and specificity indices. The agreement between fundus analyses, color images evaluation and AI analyses was quantified by the proportion of agreement (eyes in which the three-assessments overlap) and weighted thank to coefficient kappa and its 95% confidence interval. The interpretation of the kappa value was done as determined by Landis and Koch [7]: almost perfect if kappa between 0.81 and 1.00, substantial if between 0.61 and 0.80, moderate if between 0.41 and 0.60, fair if between 0.21 and 0.40, slight if between 0 and 0.20 poor if < 0. All analyses were performed using SPSS 25 (Statistical Product and Service Solutions, IBM).

## Results

We included 256 eyes of 256 consecutive patients. The mean age was 60.1 (± 16.2) years. Among these subjects 196 were diabetic and 59 not-diabetic. We excluded from statistical analysis 5 patients who presented ophthalmoscopic signs of branch retinal vein occlusion, so the final sample was of 251 eyes.

**Fig. 1:**
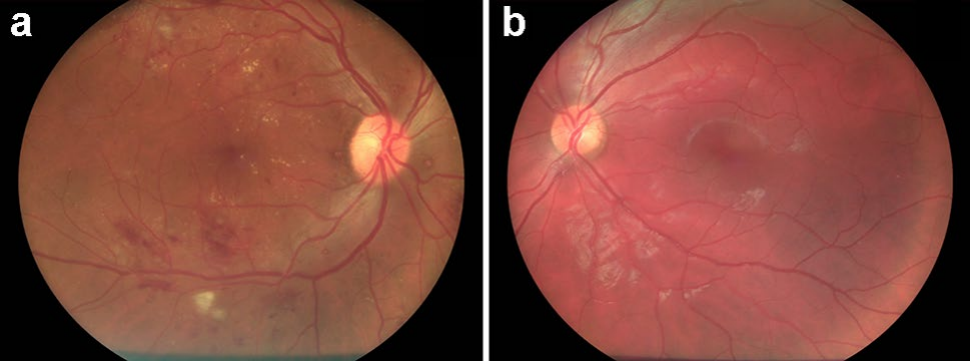
Fundus photographs (50°) of patients with (**a**) and without (**b**) signs of diabetic retinopathy acquired with the hand-held Optomed Aurora fundus camera

According to the fundus examination by a blinded retina specialist, that was considered our gold standard, 125 were diagnosed with DR and 126 without any sign of DR. The analysis of photographs obtained with the Aurora by a second blinded operator confirmed the presence of 125 subjects with signs of DR and 126 subjects without signs of DR. The agreement between these two tests was 100%. The in-built AI algorithm Selena+ identified DR in 121 patients with DR signs (Fig. 1a) and declared no-signs of DR 122 patients without DR (Fig. 1b). The four patients that were misdiagnosed by the AI presented a moderate cataract with a posterior subcapsular component.

When recognizing any sign of DR, the AI Algorithm reached a sensitivity of 96.8% and a specificity of 96.8% as reported in Table 1. The weighted kappa (95% CI) was 0.935 (0.891–0.979) showing an almost perfect agreement, according to the indication of Landis and Koch, between the AI analysis and the other two determinations (Table 2).

**Table 1:** Sensitivity and specificity of the AI algorithm for diabetic retinopathy screening

AI-based fundus examination	DR	No DR	Total
DR	96.8%	3.2%	100%
	(121)	(4)	(125)
No DR	3.2%	96.8%	100%
	(4)	(122)	(126)
Total	100.00%	100.00%	
	(125)	(126)	

*AI* artificial intelligence, *DR* diabetic retinopathy

**Table 2:** Weighted Kappa of the AI algorithm for diabetic retinopathy screening

Weighted Kappa	0.935
Standard error	0.023
95% CI	0.891–0.979

*CI* confidence interval

## Discussion

In our study we decide to test Optomed Aurora IQ camera’s performance in a situation that was as similar as possible to an extra-ophthalmological context: we didn’t use mydriatic eyedrops, we took only one macula-centered 50° photograph obtained with the auto-focus and the automated brightness adaptation and finally we decided only to discriminate the presence or absence of DR signs, as per screening purposes. On these bases we obtained an almost perfect agreement between the standard ophthalmoscopic examination and the AI algorithm sensitivity of the of 96.8% and a specificity of 96.8%. These data allow us to consider this device as an effective tool in large scale DR screening programs.

The increasing incidence of diabetes is leading DR to be an important cause of permanent vision loss worldwide, in this context, a prompt detection of the early phases of this condition with a consequent appropriate treatment is fundamental for the correct management of patients suffering for this disease [8]. Regional or local screening programs have been developed worldwide, but a DR systematic screening is complex to implement and sustain due to the lack of economic resources and a correctly coordinated heath care systems. A fixed annual screening for all people with diabetes is, for example, not sustainable if done only by a specialist ophthalmologist’s clinical examination and can rarely be shown to be cost-effective or to produce an auditable result, even if, opportunistic diabetic retinopathy screening via direct patient examinations could represent the only option in rural and remote communities [5]. Nowadays screening programs based on evaluation of color fundus photography are considered the gold standard for detecting DR having shown a better sensitivity than the ophthalmoscopic examination [5] and easier application compared to the classical seven fields approach of the Early Treatment Diabetic Retinopathy Study (ETDRS) saving time and increasing patient’s compliance [9, 10]. Unfortunately, even the evaluation of all these photographs by skilled examiners is both time and resources consuming, and can be considered a limit in a universalistic, equal and equable Health Care System. For this reason, automated image analysis and the use of AI algorithm can make an important contribution in teleophthalmology and DR screening programs.

Deep learning (DL) technology is a new branch of AI that allows software algorithm to be trained through a large mathematical function, with millions of parameters on a vast amount of data, called Convolutional Neural Network (CNN). The CNN is a deep neural network consisting in a cascade of processing layers that are inspired by human’s brain ability to learn complicated information changing the strength of synaptic connections; in DL the features are learnt by neural networks in the feature extraction stage and then used for classification [11]. DL technology is used in the Aurora AI algorithm Selena+, whose performance we decided to test in this real-world DR screening study. Another important goal in new technologies employed in DR screening is the development of handheld cameras. Traditionally fundus photographs are taken with tabletop cameras whose use limits the large-scale employment of this approach. Currently Handheld Cameras image quality seems to enable adequate gradeability [12]. Kubin et al. showed how Optomed Aurora IQ’s images had sufficient quality and high diagnostic accuracy for DR grading when compared to Canon CF-1and Zeiss Visucam 524 tabletop cameras. However, they obtained all the images in mydriasis, a condition that might substantially limit the applicability in an out-of-hospital context or in rural areas [13]. Recently a paper by Midena et al. examined the Aurora IQ’s performance in a real-life setting in a study enrolling 423 eyes, concluding that this portable fundus camera seems effective in grading any stage of DR or recognizing the presence of diabetic maculopathy [14]. Nevertheless, authors used mydriatic eyedrops before taking photographs and they didn’t exploit the in-built AI algorithm for the interpretation of results.

Our results are in agreement with those of the above-mentioned paper by Midena et al. since we showed a 100% concordance between the analysis done by the retina specialist with a biomicroscopic examination and the blinded operator who classified the images obtained with the device.

The high sensitivity (96.8%) and specificity (96.8%) of the Aurora in-built AI algorithm make our results comparable to the performance of other AI algorithms [15–19]. The IDx-DR, a FDA-approved AI algorithm, has demonstrated a sensitivity of 86.7% and a specificity of 70.0% when used in its earlier version as part of the Iowa Detection Programme [15], and, after the improvement of the system with Deep Learning feature, it changed its performance to a sensitivity of 96.8% and a specificity of 87% for the detection of DR [16]. Another AI-based approach which was used in a preliminary “disease” versus “not disease” sorting, similarly to our study, is the RetmarkerDR [17]. This software used a Machine Learning approach and revealed a sensitivity for any grade of DR of 73.0% [17]. Also Bhaskanard et al. valued the performance of another algorithm called EyeArt system v2.0 in a great sample of 107,001 cases from 404 primary care clinics reaching a sensitivity of 91.3% and a specificity of 91.1% when considering all forms of DR, and a sensitivity of 98.5% when considering treatable DR forms [18]. Another recent paper by Ruan et al. valued the sensitivity and specificity of a self-designed deep learning algorithm called Phoebus that worked using images obtained with the Aurora fundus camera [19]. It reached a sensitivity of 88.2% and a specificity of 40.7% in identifying referable forms of DR. Even if this algorithm has the advantage to separately identify different biomarkes of DR (retinal hemorrhages, hard exudation), in our opinion, for screening purposes, it is more practical to use the in-built algorithm Selena+, that we selected for our study, which also reached better values in both sensitivity and specificity. What is more, in our study, the analysis of the weighted kappa demonstrated an almost perfect agreement between the traditional screening methodology (fundus biomicroscopy and photographs analyses) and the recently introduced AI-based approaches.

In our sample, only four patients were misdiagnosed by AI, these were all phakic subjects and, in the complete examination done after the photograph acquisition, were diagnosed of a moderate cataract (3G) with significative posterior subcapsular component. We might hypothesize that in such cases a pharmacologic-induced mydriasis could help in obtaining adequate images.

For all the above-mentioned reasons we postulate that the Aurora IQ handheld camera with its AI based diagnostic algorithm Selena+ is a reliable tool that can be used not only in an ophthalmological context, but also in large scale screening programs involving primary care doctors, orthotists and nurses. This approach would contribute to relive the pressure on the ophthalmic centers that should represent the second step of the path toward a correct management of patients with sight threatening signs of DR. Furthermore, this portable device may find an important role in developing countries or in rural contexts or where the access to referral ophthalmic centers is difficult, since able to ensure a wide access to a first-line diagnostic process in DR.

We acknowledge, although in rare cases, the possibility to produce some false positive and false negative results. This bias is recognized even in clinical practice, but when considering the legal accountability in cases of misdiagnosis by an AI algorithm, it is a matter that should be clearly improved [20].

The strengths of this study are the real life setting and the context simulating an extra-ophthalmological scenario for this automated AI-based screening of DR. The main limitation of this study is the medium size of the sample. Further studies may include a wider sample and consider different disorders such as glaucoma and age-related macular degeneration [21] once the AI algorithm Selena+ will be implemented for the detection of these conditions.

In conclusion, the Optomed Aurora IQ portable fundus camera seems effective in a first-line DR screening program without use of mydriatic eyedrops. The good quality of images, the portability, and the high sensitivity and specificity of the analysis done with the in-built AI software Selena+ allow to employ this device in large scale screening programs providing an early DR detection and reducing the risk of late sight-threatening stages of the disease.
